# Consumption

**Published:** 1858-01

**Authors:** 


### CONSUMPTION.

There is no fact in pathology more certainly established, than that common consumption of the lungs can be permanently arrested, if in the advanced stages, and wholly eradicated from the system, if but in its beginnings, by the persistent habit of spending at least eight hours out of *every successive* twenty-four, in out-door activities, *regardless of all weathers.* The colder the weather, the greater the benefit will be derived from it. The worse the weather, the more necessity for being out in it; for, bad as the weather out-doors may be, that in-doors is a part of it, with the addition of all the fumes from foul cellars, dirty kitchens, and execrable water-closets.

